# Agronomic and genetic analysis of Suweon 542, a rice floury mutant line suitable for dry milling

**DOI:** 10.1186/1939-8433-6-37

**Published:** 2013-12-09

**Authors:** Young-Jun Mo, Ji-Ung Jeung, Young-Seop Shin, Chul Soo Park, Kyung-Ho Kang, Bo-Kyeong Kim

**Affiliations:** National Institute of Crop Science, Rural Development Administration, Suwon, 441-857 Republic of Korea; Department of Crop Science and Biotechnology, Chonbuk National University, Jeonju, 561-756 Republic of Korea

**Keywords:** Rice, Mutation, Sodium azide, Starch, Floury endosperm, Dry milling

## Abstract

**Background:**

Producing rice flour of good quality by dry milling is necessary to reduce milling costs and promote the processed rice food industry. This study was conducted to evaluate the dry milling properties of Suweon 542, a floury endosperm mutant, and identify the chromosomal region responsible for the floury endosperm characteristics.

**Results:**

Compared with the wild type, after dry milling process, the grain hardness of Suweon 542 was significantly lower because of its round and loosely packed starch granules. Also, the flour of Suweon 542 had significantly smaller particles and less damaged starch than Namil and other rice cultivars and its particle size distribution was similar to a commercial wheat cultivar. Considering that the yield loss of Suweon 542 due to its floury endosperm was largely compensated for by an increased number of spikelets per panicle, Suweon 542 has potential value as a raw material for rice flour production. Association analysis using 70 genome-wide SSR markers and 94 F_2_ plants derived from Suweon 542/Milyang 23 showed that markers on chromosome 5 explained a large portion of the variation in floury grains percentage (FGP). Further analysis with an increased number of SSR markers revealed that the floury endosperm of Suweon 542 was directed by a major recessive locus, *flo7*(*t*), located in the 19.33–19.86 Mbp region of chromosome 5, with RM18639 explaining 92.2% of FGP variation in the F_2_ population.

**Conclusions:**

The floury endosperm of Suweon 542 is suitable for dry milling, with a small flour particle size and low damaged starch content. Further physical mapping of *flo7*(*t*), the floury endosperm locus of Suweon 542, would facilitate efficient breeding of rice cultivars with proper dry milling adaptability that can be used in the processed rice food industry.

**Electronic supplementary material:**

The online version of this article (doi:10.1186/1939-8433-6-37) contains supplementary material, which is available to authorized users.

## Background

Rice is one of the largest crops grown in the world and is a staple food for over approximately one-half of the world population especially in Asian countries (Singh et al.^2005^). Rice is usually consumed as cooked rice and relatively a small amount is used to make ingredients for process (Zhou et al.^2002^), while recently rice flour is becoming attractive as raw material of a substitute for wheat flour in terms of ‘gluten-free’ (Demirkesen et al.^2013^), or potato-based French-fry (Kadan et al.^2001^). In Korea, only 6% of the total produced rice is used as raw materials for processed food production, although there are more than 300 types of processed food made from rice (Kim^2011^).

Rice consumption has been continuously decreasing as the eating habits of Koreans have become westernized and diversified. The per capita annual rice consumption in Korea has dropped sharply from 136.4 kg in 1970 to 69.8 kg in 2012 (Statistics Korea^2013^). The Korean government, therefore, has been trying to promote rice consumption by invigorating the processed food industry using rice flour. The status of rice consumption in Japan is also very similar to Korea, for which application of rice flour for bread making has been considered as one approach to increase rice consumption (Araki et al.^2009^).

To facilitate the market for processed rice foods, it is essential to develop proper milling technology in terms of flour particle size and damaged starch content to produce high quality rice flour at competitive cost (Hasjim et al.^2013^). Dry milling and wet milling are the two major processes used to produce rice flour. Although the dry milling process is relatively simple with a lower production cost, damaged starch content increases because of the high grain hardness of rice. In wet milling, the quality of rice flour is improved by reducing flour particle size as well as damaged starch content through soaking procedures. However, the production costs are high because of the additional expenses associated with the disposal of waste water, sterilization and drying of the wet flour (Altheide et al.^2012^; Jun et al.^2008^; Kim et al.^2009^; Lee and Kim^2011^). Recently developed technologies such as jet milling and cryogenic milling also require expensive investment and production (Ashida et al.^2010^; Tran et al.^2011^). Therefore, developing new rice cultivars with dry milling adaptability as well as good processing properties is an important goal of rice breeding in Korea.

Mutagenesis approaches using chemicals or irradiation have been widely used to diversify grain quality in rice. Satoh and Omura (1981) induced various endosperm mutants such as ‘waxy’, ‘dull’, ‘floury’, and ‘sugary’ using ethyl methanesulfonate (EMS), ethylene imine (EI), and N-methyl-N-nitrosourea (MNU). In Korea, MNU treatment of the rice variety Ilpum was successfully used by the Rural Development Administration to develop new endosperm mutant cultivars such as Seolgaeng with opaque endosperm, Baegjinju with dull endosperm, Goami 2 with resistant starch, and Keunnun with giant embryos (Hong et al.^2011^; Hong et al.^2012^,^2012^b). The Korea Atomic Energy Research Institute has released 10 rice cultivars including Nogwonchal, a glutinous cultivar, using radiation such as gamma rays (Kang et al.^2008^). Also, Shin et al. (2009) used sodium azide (SA) to develop endosperm mutant lines with waxy, dull, white-core, or floury characteristics. Among endosperm mutants such as floury, white-core or opaque kernel phenotypes, floury mutants have a white opaque grain appearance similar to waxy and dull mutants but are stained blue-black by I-KI solution and have loosely packed starch granules that break easily (Satoh and Omura^1981^). The endosperm of rice floury mutants, therefore, has more suitable physical characteristics for milling processes than normal non-glutinous lines or waxy and dull mutants in terms of the grain hardness and amylose content, respectively.

Genetic loci associated with floury endosperm in rice were previously reported on chromosomes 1, 3, 4, 5, and 8. The *flo*-*1* and *flo*-*2* mutants were induced by MNU treatment of the rice cultivar Kinmaze and their loci were localized to chromosomes 5 and 4, respectively (Satoh and Omura^1981^; Kaushik and Khush^1991^). She et al. (2010) identified the gene responsible for the floury endosperm of the *flo*-*2* mutant, *FLOURY ENDOSPERM2* (*FLO2*), which mediates protein–protein interactions. *flo*-*3* is a floury endosperm mutant with a lower level of the 16-kDa allergenic protein and its locus is on chromosome 3 (Nishio and Iida^1993^). *flo4* and *flo5* are T-DNA insertion mutant lines in *OsPPDKB* on chromosome 5 and *OsSSIIIa* on chromosome 8, respectively, that also show a floury endosperm with loosely packed starch granules (Kang et al.^2005^; Ryoo et al.^2007^). *flo*(*a*) was induced by MNU treatment of the cultivar Hwacheong and was identified as *OsTPR* on chromosome 4 (Qiao et al.^2010^). *flo6* was induced by EMS treatment of the cultivar Nipponbare and the responsible sequence variation was identified as a G/A SNP in *OsAGPL2*-*3* on chromosome 1 (Zhang et al.^2012^). Most previous studies on rice floury endosperm loci have focused on the function of identified genes while few studies have examined the practical usage of floury endosperm in terms of flour production and agricultural productivity.

In this study, we evaluated the dry milling suitability of Suweon 542 as well as its major agronomic traits related with yield potential and crop rotation systems in Korea. Genetic analysis was also carried out to identify the chromosomal location responsible for the floury endosperm characteristics of Suweon 542 to establish a basis for molecular breeding to develop rice cultivars with high quality flour.

## Results

### Agronomic traits and grain characteristics of Suweon 542

The major agronomic and yield-related traits of Suweon 542 were evaluated along with its wild type cultivar, Namil (Table 1). The heading date of Suweon 542 was 3 days later than Namil. While the culm length of Suweon 542 was 6 cm taller compared with the wild type, there was no significant difference in panicle length. Brown rice (dehulled kernel) yield per ha was 0.330 tons lower in Suweon 542 than in Namil, mainly because of the low ripened grains percentage and grain weight of the floury endosperm seeds. However, the number of spikelets per panicle was significantly increased in Suweon 542, which is likely to have compensated for a considerable amount of the yield loss caused by the floury endosperm characteristics.

**Table 1:** **Major agronomic traits of Suweon 542 in comparison with its wild type**, **Namil**

Line	HD	CL	PL	TN	SN	RGP	TGW	BRY^a^
	(days)	(cm)	(cm)	(No.)	(No.)	(%)	(g)	(ton/ha)
Namil	101^b^	78^b^	25^a^	11^a^	117^b^	87^a^	26.5^a^	6.00^a^
Suweon 542	104^a^	84^a^	26^a^	11^a^	155^a^	58^b^	22.2^b^	5.67^b^

^a^Means with the same letter are not significantly different at *P* < 0.05 in the least significant difference test (LSD_0.05_). The means of each line were obtained from replicated yield trials with three replication plots. HD: days-to-heading after sowing, CL: culm length, PL: panicle length, TN: tiller number, SN: spikelet number per panicle, RGP: ripened grains percentage, TGW: 1,000-grain weight of brown rice (dehulled kernel), BRY: brown rice yield.

**Figure 1:**
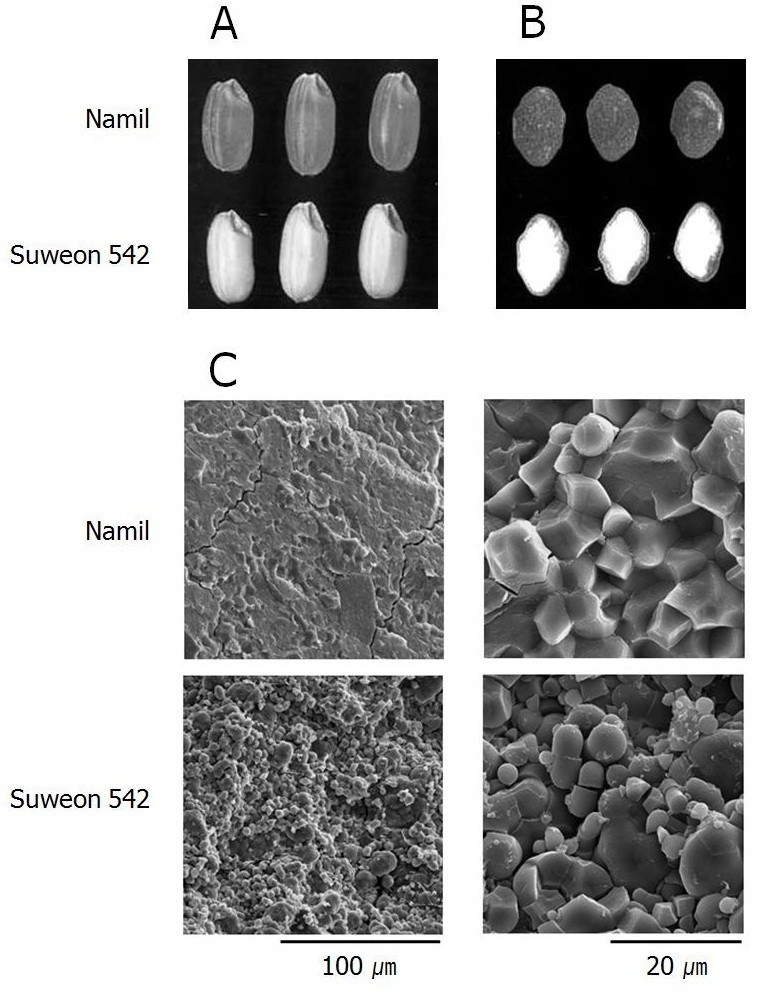
**Grain and endosperm morphology of Namil and Suweon 542.** Brown rice **(A)**, cross-section of brown rice **(B)**, and scanning electron microscope photographs of the cleaved endosperm surface **(C)**.

The grain and endosperm appearance of Suweon 542 was also examined in comparison with its wild type cultivar (Figure 1). While Namil showed transparent endosperm as a typical non-glutinous rice, the endosperm of Suweon 542 had chalky texture with white opaque appearance (Figure 1A). Cross-sectional observation of dehulled kernels revealed that most part of the Suweon 542 endosperm was white opaque except for a thin peripheral area of the grain (Figure 1B). Scanning electron microscopy showed that Suweon 542 has loosely packed starch granules with irregular and rounded shape while Namil has more densely packed starch granules with polyhedral angles (Figure 1C).

### Dry milling properties of Suweon 542

The suitability of Suweon 542 as a raw material for high quality dry milled rice flour was evaluated. The grain hardness of Suweon 542’s dehulled kernel was only about 45% of the wild type cultivar Namil (Table 2). The dehulled kernel of Suweon 542 was significantly softer than even Seolgaeng, a Korean rice cultivar with opaque endosperm, demonstrating that Suweon 542 could be easily pulverized during the milling process.

**Table 2:** **Physicochemical properties of grains and rice flours**

Line	Rice flour properties						
	Grain hardness (Kg)	Mean particle size (μm)	Damaged starch (%)	Lightness (CIE value)	Ash (%)	Protein (%)	Amylose (%)
Hwaseong	7.8 ± 1.60	112.2 ± 0.4	10.3 ± 0.2	88.6 ± 0.01	0.84 ± 0.02	7.5 ± 0.16	18.5 ± 0.2
Seolgaeng	5.9 ± 0.87	97.6 ± 1.6	7.1 ± 0.1	90.3 ± 0.06	0.72 ± 0.01	6.6 ± 0.11	17.5 ± 0.6
Namil	7.5 ± 2.21	109.1 ± 0.6	9.2 ± 0.2	88.7 ± 0.09	0.82 ± 0.01	9.2 ± 0.25	17.7 ± 0.3
Suweon 542	3.3 ± 0.48	82.0 ± 0.6	4.9 ± 0.1	90.0 ± 0.07	0.79 ± 0.01	7.5 ± 0.15	18.5 ± 0.3

Particle size and distribution in the dry milled rice flour were measured by laser diffraction analysis. The mean particle size of Suweon 542 was 82.0 μm, which was significantly smaller than Selogaeng (97.6 μm), Namil (109.1 μm) and Hwaseong (112.2 μm) (Table 2). The particle size distribution displayed in Table 3 shows that Suweon 542 had a much greater fraction of fine particles compared with the other rice cultivars. The flour particle size distribution of Suweon 542 was similar to that of Keumkang, a commercial Korean wheat cultivar, demonstrating its suitability for producing fine flour by dry milling. The damaged starch content of the dry milled flour was 4.9% in Suweon 542, which was significantly lower than in Seolgaeng (7.1%), Namil (9.2%), and Hwaseong (10.3%) (Table 2). It should be noted that damaged starch content was measured with finer flour particles in Suweon 542 compared with the other cultivars. If the three rice cultivars had been milled to a finer particle size, similar to that of Suweon 542, it is plausible that they would have had even higher damaged starch content.

**Table 3:** **Comparison of particle size distribution patterns of rice flours produced by the dry milling method**

Line^a^	Mean particle size(μm)on the relative proportions of size fraction^b^					
	Over-all	< 10%	< 25%	< 50%	< 75%	< 90%
Hwaseong	112.2 ± 0.4	45.4 ± 0.8	78.9 ± 0.6	114.1 ± 0.7	146.1 ± 0.2	174.3 ± 0.3
Seolgaeng	97.6 ± 1.6	30.7 ± 2.1	62.1 ± 2.0	98.4 ± 1.5	131.5 ± 1.3	160.0 ± 1.3
Namil	109.1 ± 0.6	44.1 ± 0.5	77.6 ± 0.7	111.6 ± 0.7	141.9 ± 0.4	168.3 ± 1.1
Suweon 542	82.0 ± 0.6	17.4 ± 0.5	38.7 ± 0.6	82.2 ± 0.6	118.6 ± 0.6	147.6 ± 1.2
Keumkang	91.0 ± 0.5	21.8 ± 0.3	48.2 ± 0.6	92.4 ± 0.4	129.6 ± 0.6	159.4 ± 1.1

^a^A Korean wheat cultivar, Keumkang, was included as a control.

^b^Standard deviations are indicated followed by mean particle size.

The flour physicochemical properties such as lightness and ash, protein and amylose contents were also measured to identify the distinct characteristics of Suweon 542 (Table 2). The flour lightness of Suweon 542 was significantly higher than Namil and Hwaseong and similar to Seolgaeng. On the other hand, the ash content of Suweon 542 was significantly lower than Namil and Hwaseong, while significantly higher than Seolgaeng. Compared with the wild type, protein content was significantly lower while amylose content was significantly higher in Suweon 542. Despite these significant differences, the overall physicochemical properties of the Suweon 542 flour were within the range of normal non-glutinous rice cultivars, except for its markedly low grain hardness due to the floury endosperm.

### Inheritance of floury endosperm in the F_2_ population

To understand the mode of inheritance of the floury endosperm characteristics of Suweon 542, a population composed of 94 F_2_ plants was developed from a cross between Suweon 542 and Milyang 23, a Tongil-type cultivar.

**Figure 2:**
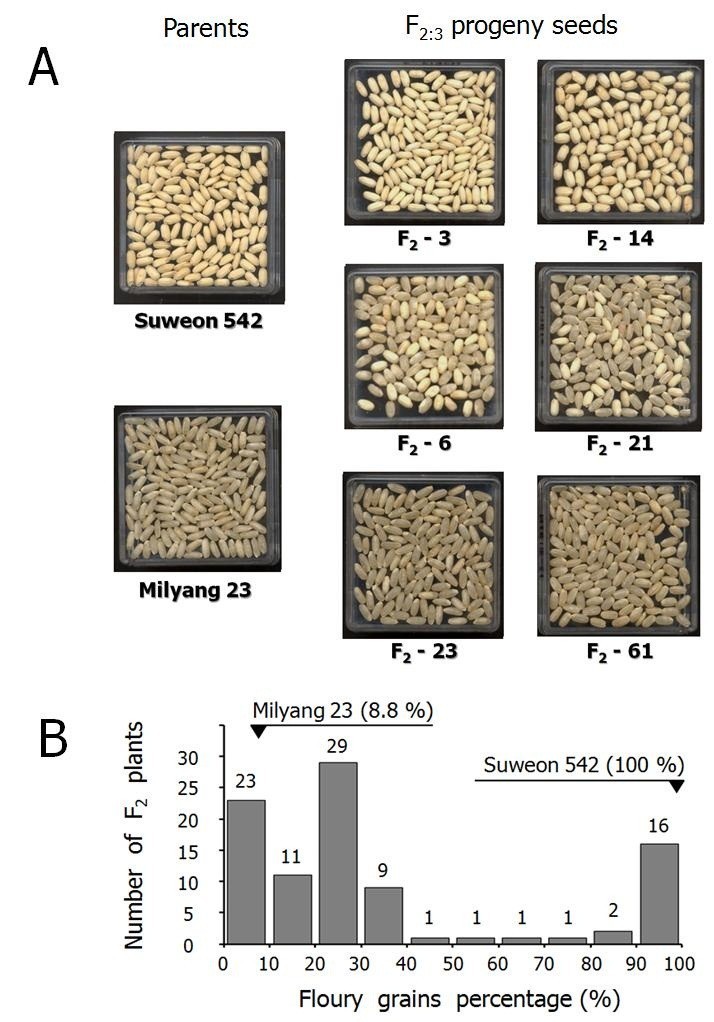
**Segregation of floury endosperm (A), and the histogram of floury grains percentage in F**_**2:3**_**seeds from the F**_**2**_**mapping population of 94 individuals of Suweon 542/** **Milyang 23 (B).** Appearance of dehulled F_2:3_ kernels from the F_2_ individuals were evaluated in terms of floury grains percentage. As examples of segregation manner within mapping population, six F_2_ progenies, homogeneous for floury endosperm (F_2_-3 and F_2_-14), heterogeneous (F_2_-6 and F_2_-21), and homogeneous for normal endosperm (F_2_-23 and F_2_-61), were presented along with their parental lines, Suweon 542 and Milyang 23.

As the phenotypic data for genetic analysis, floury grains percentage (FGP) was evaluated by using randomly selected 150–200 dehulled kernels (Figure 2A). All kernels of Suweon 542 exhibited typical floury characteristics (FGP = 100%). Grain endosperm chalkiness of rice is a varietal characteristic as some kernels of Milyang 23 had significant white opaque area (Figure 2A). Although the appearance of chalky kernel in Milyang 23 was distinguishable from floury endosperm of Suweon 542 (Figure 2A), we classified the kernels with more than 50% white opaque area as ‘floury’ during the visual inspection on the F_2:3_ seeds. Based on the criterion, the FGP of Milyang 23 was 8.8%.

We have evaluated 94 F_2_ individuals in terms of FGP by using the F_2:3_ seeds (Figure 2B). The frequency distribution of FGP exhibited an abnormal distribution pattern (skewness = 1.05, *w* = 0.97; *P* < 0.0001), with 23 individuals (24.5%) showed FGP < 10% while 16 individuals (17.0%) showed FGP > 90%. Most of the remaining 55 F2 individuals (58.5%) had FGP of 15%-35%. Therefore, it was inferred that the floury endosperm of Suweon 542 is controlled by a major recessive genetic factor. The monogenic recessive nature of floury endosperm was also strongly supported as the 55 heterogeneous F_2_ individuals segregated in a 3 normal: 1 floury ratio, where the maximum *χ*^2^ value was 4.81 (0.05 < P < 0.01). Instead of inferring genotypes of segregating progenies at the floury locus by using the estimated FGP, we conducted association analysis between the FGP and marker genotypes of F_2_ individuals by using single-locus ANOVA.

### Mapping of the floury endosperm locus in Suweon 542

A linkage map skeleton was constructed using 70 SSR markers that were polymorphic between Suweon 542 and Milyang 23 and evenly distributed across the 12 rice chromosomes, with 4–8 markers per chromosome (data not shown). Association analysis, by using single-locus ANOVA, was conducted to estimate the allelic effect of each SSR locus on the variation in FGP of the 94 F_2_ plants derived from Suweon 542/Milyang 23.

Among 6 SSR markers on the linkage map skeleton of chromosome 5 (Table 4; Locus, Ap = 1), a significant *F*-value with high *R*^*2*^ was detected at two SSR loci; RM5844 at the 9.15 Mbp region (*R*^*2*^ = 0.463) and RM3351 at the 20.76 Mbp region (*R*^*2*^ = 0.520), where the allele type of Suweon 542 was favorable to increase FGP (Table 4; Genetic effect). Although significant SSR loci were also detected on chromosomes 7 (RM1243, *R*^*2*^ = 0.079), 11 (RM5961, *R*^*2*^ = 0.064), and 12 (RM8215, *R*^*2*^ = 0.150), they were less effective than the genetic factor(s) on chromosome 5. Out of 70 SSR loci on the linkage map skeleton, related with the FGP of Milyang 23 (8.8%), RM8215 was the only locus responsible for FGP incensement by the allele type of Milyang 23 (additive effect = 4.1).

**Table 4:** **Summary of association analyses between FGP of F**_**2**:**3**_ **seeds and SSR genotypes on chromosome 5**

Locus^a^		Mirror-map information^b^				Segregation test^c^				Genotype mean^d^			ANOVA^e^		Genetic effect^f^		
Marker	Ap	Size (bp)	Mbp	BAC/PAC	cM	A	H	B	χ^2^	A	H	B	***F***	***R*** ^***2***^	Add	Dom	DeD
RM5693	1	200	0.46	OSJNBa0068N01	4.6	18	59	17	5.4	44.4	34.2	23.6	1.6				
RM3322	1	121	4.26	P0685E10	32.0	31	44	17	3.8	52.0	30.5	13.7	8.7	0.164	-19.2	-2.3	0.12
RM3193	2	126	4.97	P0453H11	36.4	29	48	17	2.6	56.3	29.3	10.4	13.6	0.231	-23.0	-4.1	0.18
RM5844	1	195	9.15	OSJNBa0032D15	53.5 ~ 54.3	30	40	24	2.4	66.3	25.7	8.1	39.3	0.463	-29.1	-11.5	0.39
RM3838	2	198	16.55	OSJNBa0077J17	60.7	28	39	26	2.0	63.0	26.3	15.4	21.5	0.323	-23.8	-12.9	0.54
RM6024	2	178	17.81	OJ1118_F06	67.5	25	41	27	1.1	75.6	26.0	7.1	70.8	0.612	-34.3	-15.3	0.45
RM18614	2	164	19.22	OSJNBa0014C03	73.9 ~ 75.0	22	51	21	0.5	81.9	27.0	1.8	101.7	0.691	-40.1	-14.9	0.37
RM164	2	265	19.26	OSJNBa0014C03	73.9 ~ 75.0	20	54	20	1.7	91.6	25.5	0.4	272.0	0.857	-45.6	-20.5	0.45
RM18620	3	245	19.28	OSJNBb0092G21	73.9 ~ 75.0	20	54	20	1.7	91.6	25.5	0.4	272.0	0.857	-45.6	-20.5	0.45
RM18624	3	304	19.33	OSJNBb0092G21	73.9 ~ 75.0	20	54	20	1.7	91.6	25.5	0.4	272.0	0.857	-45.6	-20.5	0.45
RM18639	3	317	19.73	OJ1174_H11	75.0 ~ 77.4	19	53	22	1.4	95.5	26.3	0.4	538.7	0.922	-47.6	-21.6	0.45
RM18648	3	296	19.86	P0040B10	75.0 ~ 77.4	18	53	23	1.7	95.2	27.6	1.7	290.8	0.865	-46.8	-20.9	0.45
RM1386	3	197	20.06	P0668F02	75.0 ~ 77.4	18	53	23	1.7	95.2	27.6	1.7	290.8	0.865	-46.8	-20.9	0.45
RM3351	1	134	20.76	OJ1212_B02	80.7	20	44	30	2.1	77.8	31.1	9.8	49.2	0.520	-34.0	-12.7	0.37
RM5558	2	173	21.25	OJ1301_G07	86.0	23	40	31	3.0	71.3	30.6	11.2	37.0	0.448	-30.1	-10.7	0.35
RM5642	2	129	22.25	OJ1126_D01	92.0	22	38	33	5.1	62.4	31.9	18.4	14.2	0.240	-22.0	-8.5	0.38
RM3870	2	193	22.96	OJ1387_F08	95.3	23	38	33	5.0	65.5	29.7	17.6	19.5	0.300	-24.0	-11.9	0.50
RM3476	1	132	23.91	OJ1004_E02	101.0	18	46	30	2.6	62.8	35.6	15.0	14.3	0.239	-23.9	-3.3	0.14
RM7653	2	121	27.42	OSJNBa0079H23	111.6	18	43	33	4.8	52.1	31.7	27.7	3.4	0.069	-12.2	-8.1	0.67
RM1054	1	150	29.23	OJ1007_H05	122.0	23	41	30	2.2	49.8	28.1	30.6	3.4	0.069	-9.6	-12.1	1.26

^a^DNA markers were tested in 94 F_2_ progenies derived from Suweon 542/Milyang 23. After conducting a first round of association analyses over well-defined 6 anchor markers (application: Ap = 1), an additional 9 SSR markers (Ap = 2) were applied to narrow down the putative location responsible for the floury endosperm characteristics on chromosome 5. Five SSR markers (Ap = 3) were further applied to localize the *flo7*(*t*) nearby a BAC lone OJ1174_H11 (BenBank Acc. = AC104708.2), tagged by a SSR marker, RM18639.

^b^The expected PCR product size inferred by *e*-Landings on a reference rice genome, ‘Os-Nipponbare-Reference-IRGSP-1.0’ (http://rapdb.dna.affrc.go.jp/download/%irgsp1.html). The cM position was directly adopted from the IRGSP Build5 Pseudomolecules (http://rgp.dna.affrc.go.jp/E/%IRGSP/Build5/build5.html) according to the locations of BAC/PAC clones harboring priming sites for each SSR marker.

^c^A and B are homozygous for Suweon 542 and Milyang 23 allele types, respectively, and H indicates heterozygous progenies at the tested locus. All *χ*^*2*^ values for the segregation distortion test were not significant at *P* < 0.05.

^d^Mean floury grains percentage (FGP) for each genotype category revealed by the SSR markers.

^e^Single-locus ANOVA to test associations between SSR marker genotypes and FGP. For the *F*-test, markers having less than 0.05 for significance were declared as significant empirically (^*^P < 0.05 and ^***^P < 0.001). The explainable variation portion of FGP at the tested locus (*R*^*2*^) is also shown.

^f^Additive effect (Add), dominance effect (Dom), and degree of dominance (DeD) were estimated for the significant loci: Add = (B mean – A mean) / 2, Dom = H mean – (B mean + A mean) / 2, and DeD = Dom / Add, where A and B are homozygous F_2_ individuals for Suweon 542 and Milyang 23 while H is heterozygous individuals at the tested locus.

**Figure 3:**
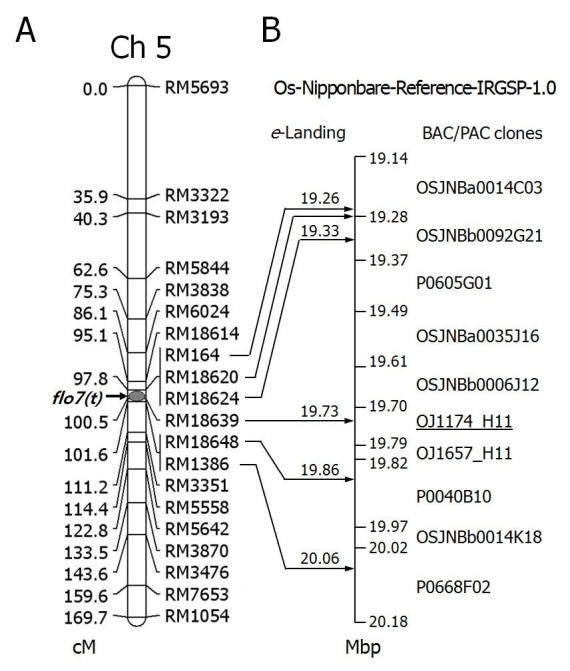
**Localization of** ***flo7*** ***(t)*** **on the linkage map of rice chromosome 5 (A), and** ***e***-**Landings of significant SSR markers on a reference rice genome to delimit the corresponding 1.04 Mbp virtual contig (B).** SSR markers were applied based on *F*-statistics from single-locus ANOVA results to narrow down the putative location responsible for the floury endosperm characteristics (see Table 4; ANOVA). The accumulative genetic distances shown in centiMorgans (cM) were calculated using the observed recombination fractions between SSR marker pairs in the F_2_ mapping population based on the Kosambi mapping function (see also Table 4; Mirror-map information). Note that the SSR markers, placed at 97.8 cM (RM164, RM18620, RM18624) and 101.6 cM (RM18648, RM1386) were co-segregated each other in the F_2_ mapping population from Suweon 542/Milyang 23 (*N* = 94). The 1.04 Mbp virtual contig, composed of overlapping 10 BAC/PAC clones, was delimited by *e*-Landings of six significant SSR markers on the reference rice genome, ‘Os-Nipponbare-Reference-IRGSP-1.0’. The underlined clone (OJ1174_H11) includes the genomic sequences corresponding to RM18639, the most significant SSR marker for the variation in FGP, and the *OsPPDKB* gene (Kang et al.^2005^).

Nine polymorphic SSR markers were applied to narrow down the putative location responsible for the floury endosperm characteristics by increasing the marker density of chromosome 5 (Table 4; Locus, Ap = 2). RM164 at the 19.26 Mbp region showed the highest *F*-value explaining 85.7% of the variation in FGP of the F_2_ population. *F*-value decreased as the SSR markers became more distant from RM164, showing 101.7 for RM18614, which was around 40 Kbp upstream of RM164, and 49.2 for RM3315, which was about 1.5 Mbp downstream of RM164 (Table 4; ANOVA). Therefore, we speculated that the floury endosperm locus of Suweon 542 was located at the region spanning RM18614-RM164-RM3351 on chromosome 5, which is at 19.22-20.76 Mbp of the reference rice genome, and 97.8-112.2 cM of linkage map constructed with the genotypes of 94 F_2_ individuals (Table 4, Figure 3A). This new recessive floury gene in Suweon 542 was tentatively designated as *floury*-*7* (*flo7*).

To construct a high density linkage map for *flo7*(*t*), 26 SSR markers only for the interval between RM164 and RM3351 were selected (data not shown). Five SSR markers (RM18620, RM18624, RM18639, RM18648, and RM1386) were positive for polymorphism between Suweon 542 and Milyang 23 and segregated for marker alleles in F_2_ progenies (Table 4; Locus, Ap = 3). However, due to the small size of mapping population (*N* = 94), RM18620 and RM18624 were mapped at the same position with RM164 (97.8 cM), and RM18648 and RM1386 also co-segregated at 101.6 cM, while RM18369 was placed at 100.5 cM (Table 4; Segregation test, Figure 3A). When association analysis was conducted again, RM18639 exhibited the highest *F* value across the chromosome 5 and explained 92.2% of the variation in FGP of the F_2_ population (Table 4; ANOVA). The result also reconfirmed that the monogenic nature of a recessive floury endosperm gene, *flo7*(*t*), which was likely to be an additive gene (degree of dominance = 0.45). The estimated additive effect of RM18639 on FGP was -47.6% (Table 4; Genetic effect), illustrating that when the genotype of Milyang 23 (*FLO7*/*FLO7*) is substituted with that of Suweon 542 (*flo7*/*flo7*), the expected FGP of Milyang 23 would be 95.2% (breeding value). Insignificant *χ2* value from the segregation test on marker genotypes of RM18639 (1.4, *P* > 0.05) also indicated that transferring the *flo7* allele into Tongil-type or *indica* rice cultivars, through conventional breeding approaches, would not be hampered by serious segregation distortion (Table 4; segregation test).

*e*-Landing of each SSR marker on the reference rice genome could confirm relative orders of the co-segregating SSR markers on a 1.04 Mbp virtual contig composed of overlapping 10 BAC/PAC clones (Figure 3B). We concluded that the intervening physical region between 19.33-19.86 Mbp of the reference rice genome harbors the major genetic factor directing the floury endosperm characteristics of Suweon 542. Compared to the adjacent markers, RM18624 (*F* = 272.0) and RM18648 (*F* = 290.8), the highest *F* value at RM18639 (538.7) strongly suggests that the most highly likely position for *flo7*(*t*) is near 19.73 Mbp region of the reference rice genome on a BAC clone OJ1174_H11 (GenBank Acc. = AC104708.2) (Figure 3B).

The ANOVA located *flo7*(*t*) in the similar chromosomal region, where *flo4* was previously reported (Kang et al.^2005^). They described that the mutant phenotype was generated by T-DNA or *Tos17* insertions into the *OsPPDKB* gene. Based on the reference rice genome database, the corresponding location was determined at 19.72 Mbp region, on a BAC clone OJ1174_H11, which also includes RM18639 - the most significant locus for the variation in FGP of the F_2_ population (Figure 3B).

## Discussion

In the present study, we examined the major agronomic traits and grain/flour physicochemical properties of Suweon 542, a floury mutant rice line suitable for dry milling, and identified the chromosomal location responsible for the floury endosperm characteristics.

The particle size and damaged starch content of grain flours are determined during the milling process. They affect flour and starch characteristics such as gelatinization and pasting properties, solubility, swelling power, and digestibility, which have great influence on the quality of processed food products (Li et al.^2013^). For rice flour, the quality of bread, noodles, and frying batters deteriorates when the raw flour has a coarse particle size and high damaged starch content (Araki et al.^2009^; Lee and Lee^2006^; Lee et al.^2013^; Heo et al.^2012^). Therefore, wet milling of polished rice has been widely used to reduce grain hardness through soaking procedures, which produces finer rice flour with less starch damage compared with dry milling. However, wet milling has a high production cost and raises environmental issues such as water waste and pollution caused by soaking procedures, hindering the expansion of the processed rice food industry in Korea. Hence, there is considerable demand for new rice cultivars suitable for dry milling.

Compared with the wild type and other rice cultivars, Suweon 542 demonstrated significantly lower grain hardness, smaller flour particle size, and lower damaged starch content when flour was produced from the dry milling of dehulled kernels (Table 2). It also showed a particle size distribution similar to that of a commercial wheat cultivar (Keumkang), which distinguished it from other rice cultivars (Table 3). These results were similar to those of Homma et al. (2007), who found that Hokuriku 166, a floury rice mutant, had finer flour than Koshihikari under both wet- and dry milling processes. Also, Ashida et al. (2010) reported that bread made from pin milled (dry milling) flour of milky-white mutants showed comparable quality to that made from jet milled (wet milling) flour of Koshihikari. Likewise, Suweon 542 could be used as a valuable breeding source for improving the quality of rice flour produced by dry milling. Polished rice is generally used to produce flour in both dry and wet milling. In this study, we demonstrated the superior flour quality of Suweon 542 when the flour was produced by dry milling of dehulled kernels without polishing. Therefore, Suweon 542 could greatly reduce milling costs by eliminating the need to polish rice grains and using existing wheat mill facilities instead of establishing separate mills for rice flour production.

Notably, the agronomic traits of Suweon 542 were also favorable for its practical use in the processed rice food industry. The yield decrease of Suweon 542 due to the low grain weight of floury endosperm seeds was largely compensated for by an increase in the number of spikelets per panicle (Table 1). In addition, Suweon 542 maintained the early maturity of the wild type, Namil, which can be used in rice-wheat double cropping systems in Korea for improved arable land use. One possible drawback of Suweon 542 is the high rate of viviparous germination under prolonged rainfall during the harvesting season (data not shown). To overcome vivipary, late-planting cultivation could be practiced to avoid the rainy period during the ripening and harvesting season. Genetically, we are crossing Suweon 542 with *indica* germplasm that has high dormancy to alleviate pre-harvest sprouting. Also, additional screening of a mutant population of Suweon 542 is being carried out to identify floury mutant lines with high sensitivity to ABA (Schramm et al.^2013^).

The observed FGP in Milyang23 (8.8%) should be considered as a varietal characteristic, because the degree of chalkiness is a complex quantitative genetic trait and it varies greatly among cultivars and among environments within a cultivar (Patindol and Wang^2003^; Liu et al.^2010^). Indeed, association analysis detected a putative minor locus on chromosome 12, RM8215 (*R*^*2*^ = 0.150), in which the allele type of Milyang 23 was favorable to increase FGP. It was expected that co-segregation of the minor locus with *flo7*(*t*) would affect the phenotype performance of the F_2_ individuals to some extent. In such case, qualitative interpretations on the phenotypes, to infer genotypes of segregating progenies at the target locus, might cause serious bias per se, due to the phenotypic mis-scoring and environmental effect (Tabien et al.^2002^). Therefore, we conducted association analysis between the FGP and marker genotypes of F_2_ individuals by conducting single-locus ANOVA, since it is less sensitive to even modest numbers of phenotypic mis-scores (Jeung et al.^2007^). Consequently, despite of the small F_2_ population size (*N* = 94), association analysis with selective markers for the putative chromosomal regions and plotting of *F* values from single-locus ANOVA provided not only estimated genetic effects but also the unbiased region of *flo7*(*t*) on chromosome 5 (Table 4, Figure 3).

On chromosome 5, *flo*-*1* and *flo4* were previously reported to control floury endosperm in rice. Although *flo*-*1* was localized to chromosome 5 by trisomic analysis, no information is available giving a more detailed location (Satoh and Omura^1981^; Kaushik and Khush^1991^). The floury endosperm mutant, *flo4* was generated by knockout mutations in the C_4_-type pyruvate orthophosphate dikinase gene (*OsPPDKB*) (Kang et al.^2005^), and locates within a BAC clone OJ1174_H11 (Figure 3B). By using the 94 F_2_ individuals, in the present study, the most highly likely region for *flo7*(*t*) was determined on chromosome 5 as the 3.8 cM interval (97.8-100.6 cM; Figure 3A), which is corresponding to the 530 Kbp physical region (19.33-19.86 Mbp; Figure 3B). The 530 Kbp physical region includes the BAC clone OJ1174_H11 harboring the most significant locus, RM18639, for the variation in FGP of the F_2_ population (Table 4; ANOVA). The result strongly suggests that *flo7*(*t*) is physically linked to *flo4*, or might be another mutated allele of the *OsPPDKB* gene.

Kang et al. (2005) described the mutant phenotype of *flo4* as ‘white-core’ endosperm with a normal outer portion. As the determined effects of the *flo4* mutant on rice endosperm, they also characterized the aberrant physicochemical properties as 1) reduced kernel weight, 2) decreased amylose content, 3) increased protein content, and 4) significantly increased total lipid content. Unlike their description, Suweon 542 had an entirely white opaque kernel except for a thin peripheral area (Figure 1B). Moreover, Suweon 542 was significantly different from Namil (wild type) in terms of reduced protein content and increased amylose content, while the reduced kernel weight was consisted with *flo4* (Table 2). Ashida et al. (2009) classified chalky rice mutant lines into ‘white-core’, having an opaque area only in the central region of the grain, and ‘milky-white’, with an entirely opaque grain except for the peripheral area, and observed that, when dry milled, the milky-white lines had significantly smaller flour particle size and lower damaged starch content compared with the white-core lines. While *flo4* had white-core characteristics with a central chalky area (Kang et al.^2005^), Suweon 542 showed milky-white characteristics with an overall opaque endosperm, so might be more suitable for dry milling (Figure 1, Table 3).

To efficiently transfer the floury endosperm characteristics of Suweon 542 to other commercial rice cultivars, it is essential to develop DNA marker tightly linked to the target gene, *flo7*(*t*). Because Suweon 542 is a mutant line induced by sodium azide (Shin et al.^2009^), it is desirable that the DNA marker depends on the authentic DNA polymorphism between Suweon 542 and Namil (wild type) followed by 1) fine physical mapping for *FLO7* locus, 2) map-based cloning of the gene responsible for *flo7* mutant, and 3) verifying the gene through complementing *flo7* mutant line with transgenic line harboring a complete wild-type copy of *FLO7*. Currently, by using F_2:3_ progeny lines derived from the heterozygous F_2_ individuals at RM18639, construction of a segregating population is in progress to conduct fine mapping of *flo7* locus, as well as to verify whether *flo7*(*t*) is another mutated allele of *OsPPDKB* as *flo4* or a different gene with close proximity to *OsPPDKB*.

## Conclusions

The floury endosperm of Suweon 542 makes it suitable for dry milling with a small flour particle size and low damaged starch content. Through genetic analysis on the F_2_ progenies derived from Suweon 542/Milyang 23, a floury endosperm locus, *flo7*(*t*), was located on chromosome 5 as a major recessive genetic factor directing floury endosperm of Suweon 542. Further physical mapping of *flo7*(*t*) would facilitate efficient breeding of rice cultivars with proper dry milling adaptability that can be used in the processed rice food industry. Based on our studies, Suweon 542 was patented in Korea with its designated name, ‘Namil(SA)-flo2’ (Jeung et al.^2012^).

## Methods

### Plant materials and DNA extraction

Suweon 542 is a floury endosperm mutant line derived from sodium azide treatment on a high-yield, early maturing, and non-glutinous *japonica* rice cultivar, Namil (Shin et al.^2009^). In brief, after harvesting a single panicle from each M_1_ plant, the ear-to-row method was used to advance generations with fertile individuals until the M_7_ generation. A total of 5,135 lines (M_8_) with homogeneous phenotypes were eventually established as a mutant stock of Namil. To identify floury mutants with white opaque endosperm that crumble easily into a fine powder (Kaushik and Khush^1991^), dehulled kernels (brown rice) were screened followed by I-KI staining to exclude waxy and dull mutants. Out of three floury mutants, the line with the pedigree ‘Namil(SA)M2-1509-RGA-1-1-1-1’, was designated as ‘Namil(SA)-flo2’. After conducting local adaptation tests, the mutant line was also registered, as a Korean elite rice line, ‘Suweon 542’.

To evaluate major agronomic traits and grain/flour physicochemical properties, Suweon 542 was cultivated with Namil (wild type, non-glutinous *japonica* cultivar), Hwaseong (non-glutinous *japonica* cultivar), and Seolgaeng (non-glutinous opaque endosperm *japonica*; Hong et al.^2011^) in the experimental plot of the National Institute of Crop Science (NICS), Rural Development Administration (RDA), Suwon, Korea. During dry milling suitability tests, the harvesting of a Korean wheat cultivar, Keumkang (Park et al.^2011^), was included as a control.

A total of 94 F_2_ progenies from a cross between Suweon 542 and Milyang 23, a non-glutinous Tongil-type (derived from an *indica*-*japonica* cross) cultivar, were used for genetic studies on the induced floury endosperm characteristics. The parents and their F_2_ progenies were grown by conventional methods in the experimental plot of the NICS, RDA. Total genomic DNA was extracted from fresh leaf tissue of the filed-grown F_2_ and parental lines according to Murray and Thompson (1980), with minor modifications.

### Evaluation of agronomic traits and grain/flour physicochemical properties

Replicated yield trials (RYT) were conducted to evaluate major agronomic traits as well as grain/flour physicochemical properties in the field at NICS, RDA, Suwon, Korea in 2010. The seeds of each rice line were sown on April 25, and were transplanted on May 25 under a randomized complete block design (RCBD) with three replication plots. Each plot, consisting of eight rows with 30 hills per row and three plants per hill, was planted with 30 × 15 cm spacing. Rice lines were nursed and evaluated for major agronomic traits following the standard evaluation method for rice (RDA, Korea^2003^). The amount of fertilizer application was 90-45-57 Kg/ha for N-P_2_O_5_-K_2_O, and the 10 hills in the middle rows were used to determine days-to-heading (HD), culm length (CL), panicle length (PL), tiller number (TN), spikelet number per panicle (SN), and ripened grains percentage (RGP). Grain yield per plot was evaluated based on a grain harvest of 100 hills in the central row of each plot. Brown rice (dehulled kernels) yield (BRY; ton/ha) was then calculated based on the brown rice recovery rate. The 1,000-grain weight (TGW) was measured in grams as the average weight of 1,000 fully filled brown rice grains from each plot.

The grain hardness of brown rice was determined by measuring the pressure at grain breakage point using a 5-mm probe attachment of TA.XT Plus (Stable Micro Systems, Godalming, Surrey, UK) under the conditions of 0.4 mm/sec and 40.0 g for test speed and trigger force, respectively. The cleaved endosperm surface was observed with an S-550 field emission scanning electron microscope (Hitachi Hi-Tech, Tokyo, Japan).

After tempering, the dehulled kernels of Suweon 542 and other rice cultivars were milled using a Buhler MLU-202 laboratory mill (Buhler AG, Uzwil, Switzerland) with Keumkang, a Korean wheat cultivar, as a control. An extraction of about 60% was prepared by blending the first and second break millstreams and the first and second reduction millstreams to examine physicochemical properties. Particle size distribution in the flour was determined using a LS13320 laser diffraction particle size analyzer (Beckman Coulter, CA, USA). Five grams of flour was transferred into the laser diffraction particle size analyzer’s dispersion tube for size measurement. Damaged starch content was evaluated with a starch damage assay kit (Megazyme International Ireland, Wicklow, Ireland) following the manufacturer’s instructions. The lightness of rice flour was measured with a JS-555 (Color Techno System, Tokyo, Japan). Moisture, protein, and ash contents of the rice flour were determined following the 44-15A, 46–30, and 08–01 methods of AACC (AACC American Association of Cereal Chemists^2000^). The amylose content of the rice flour was estimated by the method of Juliano (1971).

### Genetic studies

Dehulled kernels of parental lines and F_2:3_ seeds harvested from the 94 F_2_ individuals were investigated in terms of floury grains percentage (FGP), as the phenotypic data for genetic analysis. Randomly selected 150–200 seeds were dehulled to evaluate FGP by visual inspection, where a dehulled kernel with more than 50% opaque area was considered as ‘floury’

A total of 70 SSR markers displaying polymorphism between Suweon 542 and Milyang 23 were selected as anchor markers after careful comparisons with previous reports in terms of the band size of amplified products (McCouch et al.^2002^). The F_2_ mapping population of 94 individuals was genotyped at the loci tagged by the preselected anchor markers to construct a linkage map skeleton. To determine the relative physical positions of the SSR markers, their primer sequences were projected over a reference rice genome (*e*; Jeung et al.^2007^), Os-Nipponbare-Reference-IRGSP-1.0 (http://rapdb.dna.affrc.go.jp%/download/irgsp1.html), using the ‘BLAST’ menu at the Rice Annotation Project database (RAP-DB) (http://rapdb.dna.affrc.go.jp/tools/blast). When both primer sequences successfully recognized their physical locations for annealing, the expected PCR product size was calculated to judge the PCR products. Based on the defined physical locations, the corresponding BAC/PAC clones were identified and their determined cM positions were directly adopted from the IRGSP Build5 Pseudomolecules (http://rgp.dna.affrc.go.jp/%E/IRGSP/Build5/%build5.html). After identifying rice chromosome 5 as the location of the most significant genetic factor for floury endosperm characteristics, 14 additional SSR markers were further incorporated into the linkage map skeleton to increase marker density (see Table 4; Locus, Ap = 2, 3).

PCR was performed in a total volume of 30 μl containing 10 ng of DNA template, 10 pmol of each primer, 1.5 mM of MgCl_2_, 0.2 mM of dNTPs, and 1 U of *Taq* polymerase (Nurotics, Daejeon, Korea). PCR began with one cycle at 95°C for 3 min, followed by 40 cycles of 95°C for 30 s, 55°C for 30 s, and 72°C for 1 min, with a final extension at 72°C for 10 min (MJ Research PTC-100 thermocycler; Waltham, MA, USA). The primer annealing temperature for SSR markers was 55°C. Sequencing gel electrophoresis (5% polyacrylamide, 6 M urea, 1 × TBE, 80 W) was used for SSR-PCR product separation and bands were visualized using Silver Sequence™ (Promega, Madison, WI, USA).

Chi-square analysis was conducted for segregation tests on the observed number of floury kernels and the revealed genotypes of F_2_ individuals for each SSR locus. PROC UNIVARIATE and PROC GLM of the SAS statistical package (Version 8.1, SAS institute, Cary, NC, USA) were used to calculate descriptive statistics on FGP of the F_2:3_ seeds, and to estimate the relative contribution of tested loci to floury grains percentage (single-locus ANOVA), respectively. For the *F*-test of loci, markers having a *P* value less than 0.05 were declared to be significant empirically. The percentage of phenotypic variation explained (*R*^*2*^), additive genetic effect, and the degree of dominance were then estimated for the declared loci within the mapping population. Judging the predetermined SSR marker orders, through *e*-Landing, on the linkage map of chromosome 5, and estimating the practical recombination fractions between SSR marker pairs were conducted with the ‘ripple’ and ‘map’ commands of the computer software MAPMAKER/EXP3.0 (Lincoln et al.^1992^). The marker intervals were calculated by using the Kosambi mapping function.

## Authors’ original submitted files for images

Below are the links to the authors’ original submitted files for images. Authors’ original file for figure 1 Authors’ original file for figure 2 Authors’ original file for figure 3
