# Supplementary figures and images for: Agronomic and genetic analysis of Suweon 542, a rice floury mutant line suitable for dry milling
Source: Rice (N Y). 2013 Dec 9;6:37. doi: 10.1186/1939-8433-6-37 (PMC4883716; doi:10.1186/1939-8433-6-37)

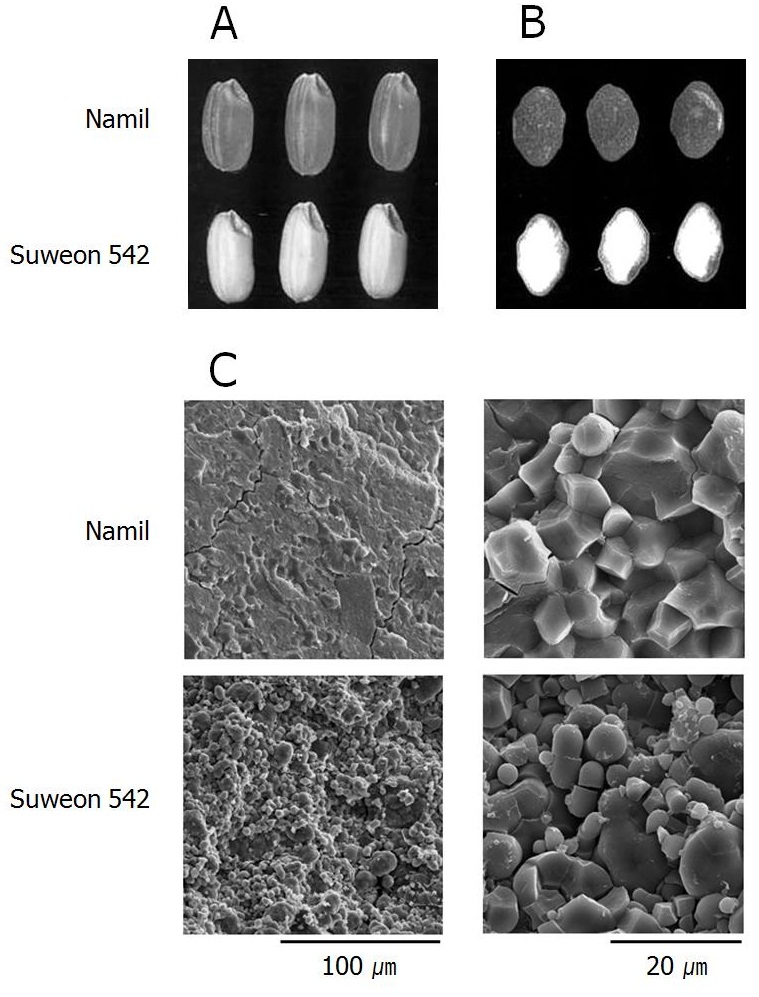

Supplement: Supplementary file 1 — Authors’ original file for figure 1 [file 12284_2013_70_MOESM1_ESM.jpeg]

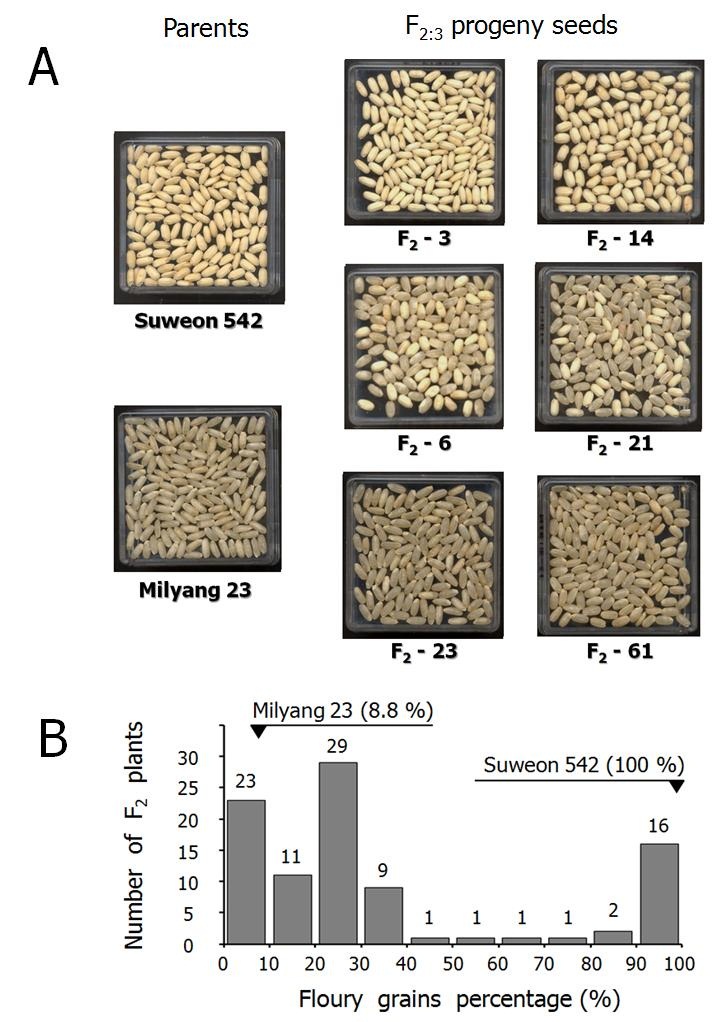

Supplement: Supplementary file 2 — Authors’ original file for figure 2 [file 12284_2013_70_MOESM2_ESM.jpeg]

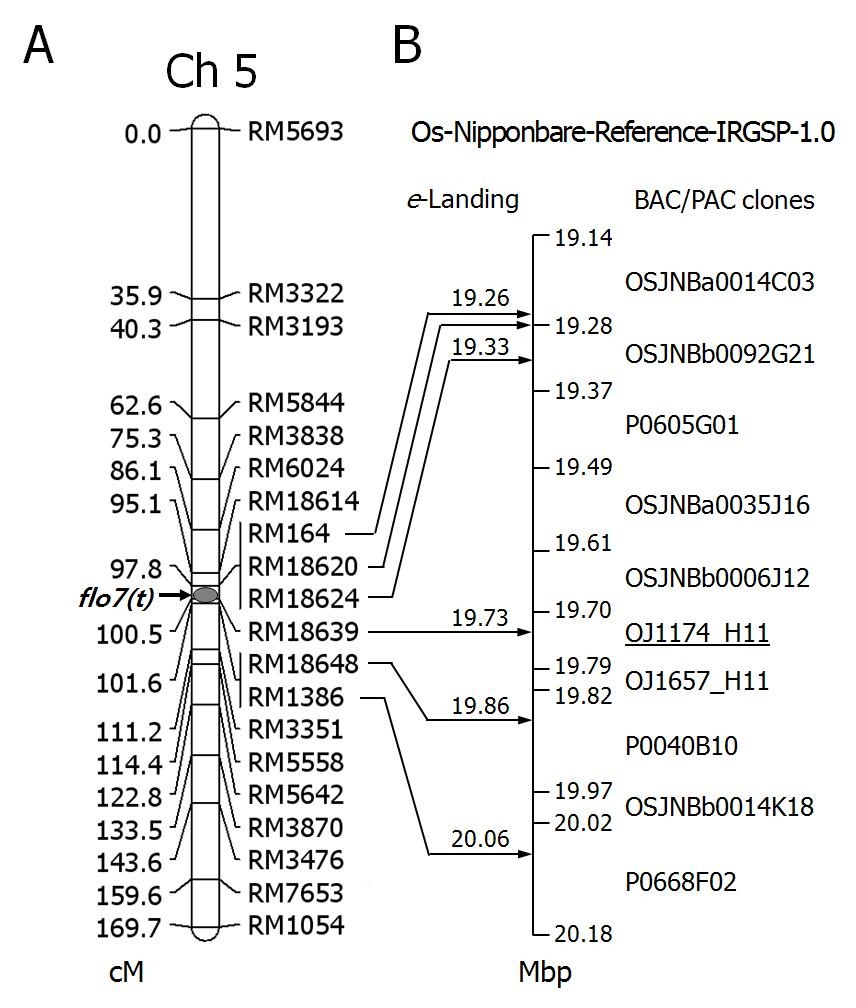

Supplement: Supplementary file 3 — Authors’ original file for figure 3 [file 12284_2013_70_MOESM3_ESM.jpeg]
